# Un mode de révélation rare de la maladie de Wegener: une myocardite associée a une endocardite fibroblastique

**DOI:** 10.11604/pamj.2016.23.133.8323

**Published:** 2016-03-25

**Authors:** Salim Arous, Ilham Bensahi, Malika Noureddine, Rachida Habbal

**Affiliations:** 1 Service de Cardiologie, CHU Ibn Rochd, Casablanca, Maroc

**Keywords:** Granulomatose de Wegener, endocardite fibroblastique, Myocardite, Flutter auriculaire, Wegener's granulomatosis, fibroplastic endocarditis, myocarditis, atrial flutter

## Abstract

Nous rapportons à travers cette observation le cas rare d'une maladie de Wegener révélée par une myocardite associée à une endocardite fibroblastique. Le patient a été admis initialement dans un tableau d'insuffisance cardiaque globale, avec un trouble du rythme type flutter auriculaire à l'ECG. A l’échocardiographie le ventricule gauche était non dilaté, siège d'une dysfonction sévère, avec un dosage des troponines positif. Une insuffisance rénale sévère a été découverte fortuitement nécessitant une hémodialyse, associée à une anémie inflammatoire confirmée par la férritinémie et le myélogramme. Le dosage des c-ANCA était fortement positif confirmant le diagnostic. La TDM thoracique avait objectivé une pneumopathie basale droite. Après avoir démarré un traitement adapté comprenant une corticothérapie et un traitement immunosuppresseur, l’évolution a été favorable avec normalisation de la fonction systolique du ventricule gauche. Bien que les manifestations cardiaques cliniques évidentes soient rares, l'atteinte cardiaque au cours de la maladie de Wegener est décrite, nécessitant une orientation diagnostic rapide et une connaissance rigoureuse de cette maladie grave.

## Abstract (en)

We report, through this observation, a rare case of Wegener's disease revealed by myocarditis associated with fibroblastic endocarditis. The patient was initially admitted to hospital with global heart failure. The ECG showed atrial flutter rhythm disorder. Echocardiographic examination revealed non-dilated left ventricle with severe dysfunction and positive troponin dosage. Severe renal failure was discovered fortuitously and required hemodialysis. It was associated with an inflammatory anaemia confirmed by serum ferritin and myelogram. c-ANCA dosage was strongly positive, confirming the diagnosis. Chest CT scanning objectified right basal pneumonia. After starting appropriate treatment, including corticotherapy and immunosuppressive treatment, evolution was favorable with the normalization of left ventricular systolic function. Although obvious clinical cardiac events are rare, cardiac involvement in Wegener's disease is described, requiring rapid diagnostic orientation and rigorous knowledge of this serious disease.

## Introduction

La granulomatose de Wegener (GW) est une vascularite nécrosante systémique caractérisée par la formation de granulomes, qui affecte le plus souvent les voies respiratoires et les reins [1]. L'atteinte cardiaque au cours de la maladie de Wegener est rare, dont la fréquence anatomique est de l'ordre de 25% dans les séries autopsiques [2]. Cette fréquence ne concorde pas avec celle rapportée dans les études cliniques, inférieure à 8% [3]. Elle s'associe à un pronostic réservé particulièrement si le diagnostic a été tardif. Un large éventail d'anomalies cardiaques a été décrit incluant la péricardite, les lésions valvulaires, la coronarite, la myocardite et les troubles du rythme cardiaque. Un seul cas d'endocardite fibroblastique compliquant la maladie de Wegener a été rapporté, révélé lors d'une autopsie. L'intérêt de cette observation réside dans la rareté du tableau menant au diagnostic de la maladie de Wegener, comprenant une atteinte des trois tuniques (endocarde, myocarde et péricarde) avec un trouble du rythme supra-ventriculaire.

## Patient et observation

**Figure 1:**
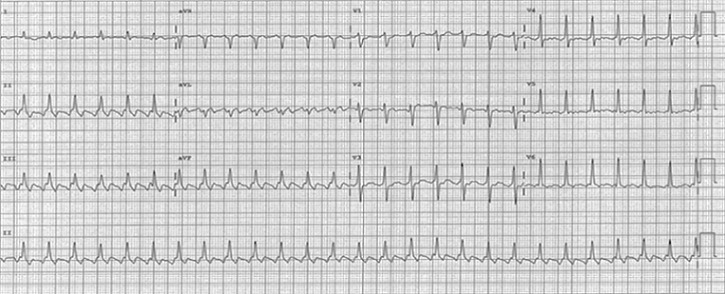
ECG montrant un Flutter à conduction 2/1, avec une réponse ventriculaire à 150bpm

**Figure 2:**
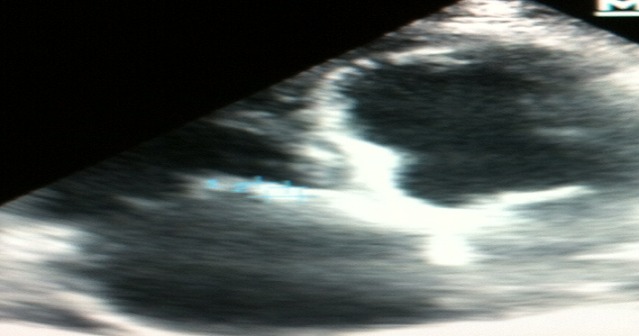
Végétation de 8/6mm sur le feuillet septal antérieur de la valve tricuspide

Nous rapportons l'observation d'un homme âgé de 50 ans, sans facteur de risque cardiovasculaire, suivi depuis deux mois avant son hospitalisation pour une anémie inflammatoire d'origine indéterminée nécessitant plusieurs transfusions, admis dans notre unité de soins intensifs pour un tableau d'insuffisance cardiaque globale avec des râles crépitant dépassant les mi-champs pulmonaires, des œdèmes des membres inférieurs et une turgescence des veines jugulaires, sans notion de douleurs thoraciques, le tout dans un contexte d'apyrexie et d'altération de l’état général. À l’électrocardiogramme avait révélé un trouble du rythme cardiaque supra-ventriculaire type flutter auriculaire à conduction 2/1 (Figure 1). A l’échocardiographie le ventricule gauche était non dilaté, avec une dysfonction sévère systolique bi-ventriculaire, la fraction d’éjection (FEVG) a été estimée à 25% en Simpson biplan, des pressions de remplissages du ventricule gauche élevées, une insuffisance tricuspide modérée avec une HTAP à 68mmHg et une végétation de 8/6mm sur le versant auriculaire du feuillet septal antérieur de la valve tricuspide avec un épanchement péricardique de faible abondance (Figure 2). Sur le plan biologique, le bilan inflammatoire était perturbé avec une CRP à 86 mg /l et une VS positive à 50 mm, par contre le bilan infectieux comprenant des hémocultures et un ECBU, était négatif. Le dosage des troponines était positif à 4,2 ng/ml avec une insuffisance rénale sévère, le débit de filtration glomérulaire calculé par MDRD était à 9ml/min/1.73m^2^, et une protéinurie positive à 1600mg/24h. Le taux d'hémoglobine était à 8g/dl, l'origine inflammatoire a été confirmée par le dosage de la férritinémie et le myélogramme.

Devant ce tableau associant une atteinte cardiaque, rénale et hématologique, nous avons fortement suspecté une maladie de système ou une vascularite. Le reste des investigations a montré un taux des AAN limite avec un aspect moucheté (80 pour un seuil de 80), et le dosage des c-ANCA était fortement positif à un titre de 76 UI/l, de spécifité anti-PR3 confirmant le diagnostic de la maladie de Wegener. La biopsie rénale était peu contributive, ne montrant pas l'aspect de granulome, mais retrouvant l'aspect d'inflammation vasculaire et une destruction glomérulaire importante. L'examen ORL était strictement normal. La TDM thoracique avait objectivé une pneumopathie basale droite sans nodule individualisé. La coronarographie n’était pas réalisée vu l'altération de la fonction rénale. Devant le rattachement de l'atteinte cardiaque à la maladie de Wegener, l'IRM cardiaque n'a pas été demandée. Initialement le patient a bénéficié d'une séance d'hémodialyse et une transfusion de deux culots globulaires avec un traitement symptomatique comportant le furosémide injectable, une faible dose d'IEC, la Cordarone par voie intraveineuse et un traitement anticoagulant. Une antibiothérapie par voie intraveineuse à base de Ceftriaxone pendant quatre semaines. Après stabilisation, une faible dose de béta bloquant a été initiée avec augmentation progressive des doses. Le traitement d'attaque consistait en un traitement corticoïdes intraveineux puis oral à 1 mg/kg et des bolus de cyclophosphamide intraveineux, permettant une rémission clinique et biologique 4 mois après l'initiation du traitement, une réduction spontanée du flutter avec normalisation de la fonction systolique du ventricule gauche, FEVG contrôlée à 50% avec une fonction ventriculaire droite normalisée et une réduction importante de l'hypertension pulmonaire, contrôlée à 38 mmHg.

## Discussion

Dans cette observation, notre patient avait une maladie de Wegener avec atteinte rénale, pulmonaire et cardiaque type trouble du rythme supraventriculaire, myocardite et endocardite fibroblastique. Forstot et al. retrouvent dans leur étude rétrospective comprenant 27 patients avec granulomatose de Wegener et atteinte cardiaque, qu'il s'agit de péricardites dans 50% des cas, de coronarites dans 50% des cas, de myocardites dans 25% des cas, de valvulopathies ou d'endocardites dans 21% des cas, de troubles de conduction dans 17% des cas et d'infarctus du myocarde dans 11% des cas [4]. Les myocardites sont dues à l'atteinte granulomateuse du myocarde, elles peuvent être diagnostiquées sur un épisode de décompensation cardiaque ou de précordialgie, ou être asymptomatique mais évoluer lentement vers une cardiomyopathie dilatée. Les coronarites sont liées à des sténoses inflammatoires des artères de moyen et petit calibre, qui restent le plus souvent asymptomatiques mais peuvent évoluer vers l'infarctus du myocarde et le décès. L'atteinte valvulaire peut être primaire, liée à une inflammation des tissus, ou secondaire à une endocardite. Les arythmies atriales et ventriculaires sont le symptôme d'une anomalie sous-jacente à rechercher: péricardite, infarctus ou encore endocardite [5]. Un seul cas d'endocardite fibroblastique sur maladie de Wegener a été décrit dans la littérature, découverte lors d'une autopsie [6]. Des cas d'atteinte valvulaire type rétrécissement mitral ont été rapportés [7]. L'atteinte cardiaque est reconnue comme un facteur de mauvais pronostic d'une GW notamment parce qu'elle témoigne le plus souvent d'une atteinte systémique diffuse de la vascularite. Une étude rétrospective sur 108 patients atteints de GW avec complications rénales retrouvait un taux de mortalité globale de 24%. Trente-cinq pour cent de ces décès étaient secondaires à une atteinte cardiaque (infarctus du myocarde ou mort subite) [8]. L'examen anatomopathologique permet de confirmer le diagnostic, objectivant des lésions de vascularite nécrosante ou des granulomes. L'IRM semble en effet plus sensible que l’échocardiographie pour le diagnostic et le suivi de l'atteinte myocardique inflammatoire liée à la GW, ce qui permettrait de guider les décisions thérapeutiques [9]. Le dosage biologique des c-ANCA a rapporté un apport considérable dans le diagnostic et le traitement de la granulomatose de Wegener. Le pronostic peut être nettement amélioré par un traitement adéquat et précoce par les corticostéroïdes et les immunosuppresseurs.

## Conclusion

L'atteinte cardiaque au cours de la maladie de Wegener est rare, nécessitant une orientation diagnostic rapide et une connaissance rigoureuse de cette maladie grave. Elle peut être la conséquence de la vascularite ou de l'atteinte granulomateuse, et doit être si possible confirmée par une analyse anatomopathologique. La présence d'une cardiopathie même infraclinique est un facteur de mauvais pronostic.
